# Metamaterial Incident Photon Reconstruction Theory Based on Resonant Dipole Phase

**DOI:** 10.3390/mi17010130

**Published:** 2026-01-20

**Authors:** Boli Xu, Renbin Zhong

**Affiliations:** Terahertz Research Center, School of Electronic Science and Engineering, University of Electronic Science and Technology of China, Cooperative Innovation Centre of Terahertz Science, Chengdu 610054, China; 202211022430@std.uestc.edu.cn

**Keywords:** metamaterial, amplitude–phase relationship, resonant phase

## Abstract

In this study, a Metamaterial Incident Photon Reconstruction Theory (MIPRT) is developed to describe the modulation process of metamaterials on incident photons. The theory includes the Invariant Incident Photon Hypothesis and Resonant Phase Deconstruction and Quantification; it reveals the modulation characteristics of metamaterials on incident photons, not by first absorption and then re-emission but by inducing coherent destructive interference, which brings about redistribution of the spatial probability of photon occurrence. This theory is validated in a single-layer metamaterial, and a unique relationship between the resonant phase and amplitude is derived and confirmed by simulation. The proposed MIPRT brings a comprehensive understanding of the electromagnetic (EM) response characteristics of metamaterials and provides a new idea for metamaterial theory from another perspective.

## 1. Introduction

Metamaterials designed to control the amplitude, phase, or wavefront of electromagnetic (EM) waves have been widely studied in recent years [1,2,3]. Researchers have proposed various theories to reveal the laws underlying the phenomenon of the controlling of EM waves by metamaterials. The principle of multilayer metamaterials modulating phenomena is well described by transmission matrix method theory [4]. The theory of the photon band diagram can interpret the characteristics of EM wave transmission inside metamaterials [5], and these kinds of metamaterials are also called photonic crystals occasionally. Considering the surface phase gradient distribution, the generalized Snell equations describe the formation of the EM wavefront controlled by metamaterials [6]. Ahmed H. et al. used two-port equivalence to derive the relationship of transmission phase limitations by mathematical reasoning [7]. These methods rely heavily on numerical simulations but lack insight into the physical mechanisms. As for the analytical methods, such as impedance analysis [8], transmission line equivalence [9], and general dipole analysis [10,11], they hold significant practical value in the study of metamaterials, especially in design, optimization, and engineering applications.

In this paper, with the Invariant Incident Photon Hypothesis, the MIPRT theory is proposed with the definition of the plane wave phase (PWP) and resonant dipole phase (RDP); MIPRT outlines the modulation process of metamaterials’ EM waves via resonant dipoles. The analytical relationship between the resonance phase and the amplitude of the reflected wave is derived for a single-layer metamaterial, and the results are verified through simulation. It provides a deeper understanding of the electromagnetic response characteristics of metamaterials, which could play a key role in the design of artificial materials. Particularly in the context of micro- and nanoscale device design, the modulation processes and underlying regularities revealed in this work provide clear directional guidance for the analysis and design of target devices. In addition, the explicit relationship between amplitude and phase modulation offers an intuitive constraint on admissible parameter spaces, thereby helping designers avoid physically unattainable device configurations.

## 2. Metamaterial Incident Photon Reconstruction Theory

### 2.1. The Invariant Incident Photon Hypothesis

The model of the resonant dipole is commonly adopted to qualitatively explain the interaction of metamaterials and electromagnetic waves (incident photons). The interaction between the metamaterial and the incident photons includes two steps: the excitation of resonant dipoles in the metamaterial and the modulation of the incident photons by the metamaterial. Once the interaction reaches a steady state, the latter step of modulation of the incident photons becomes the dominant effect. At this stage, previous theories usually suggested that incident photons are firstly absorbed by resonant dipoles upon striking the metamaterial, after which the dipoles emit new photons. However, the Invariant Incident Photon Hypothesis asserts that incident photons are not absorbed by resonant dipoles, but instead undergo photon interference with the photons emitted by the dipoles, resulting in a new spatial probability distribution of the photons. Therefore, there are invariant incident photons in the whole space, including the incident half space and the transmission half space separated by the metamaterial. The new spatial probability distribution of photons is described as the probability amplitude $A_{new}$ in both half spaces:

(1) $$\begin{array}{c} A_{new}=A_{i}+A_{s} \end{array}$$

Here, $A_{i}$ represents the probability amplitude of incident photons (IPs) and $A_{s}$ is the probability amplitude of resonant dipole-emitted photons (RPs). Thus, the RPs correspond to the probability amplitude of reflected photons in the incident space, while in the transmission space, the direction of momentum of RPs is same as that of IPs, so the $A_{new}$ of IPs and RPs in the transmission space represents the probability amplitude of the photons modified by the metamaterial.

From the perspective of energy conservation, the coherent destructive interference between IPs and RPs leads to a reduction in energy in the transmission space. This energy decrease is just equal to the RP energy in the incident space, which is considered as an energy increase. In other words, the incident photons and dipole-emitted photons undergo coherent destructive interference, resulting in a new spatial distribution of photons. Therefore, the spatial distribution probability of the modulated photons decreases in the transmission half space and increases in the reflection half space. During the modulation process, the incident photons remain invariant throughout the entire space rather than being absorbed by the resonant dipoles.

### 2.2. Resonant Phase Deconstruction and Quantification

The key parameters of a photon are amplitude and phase: amplitude represents energy, and phase differences of the coherent photons will bring about destructive interference and then govern the conservation of energy. The resonant dipole produces near-field photons (localized EM field) and far-field photons (radiated field). Near-field photons are spatially confined around the dipole, while far-field photons propagate outward, carrying energy and phase information to the far field. Therefore, to discuss the modulation process in detail, we define the phase associated with near-field photons to be the resonant dipole phase (RDP, related to the near-field radiation pattern of resonant dipoles), while the phase of far-field photons is defined as the plane wave phase (PWP, representing the far-field radiation pattern of resonant dipoles). To facilitate calculation, the incident photon phase is taken as the zero reference phase; then the PWP and RDP can be deduced.

We take a single-layer metamaterial as a case; it has strip-shaped units, with a strip length of 50 μm and a period of 80 μm, and its resonance frequency is 2.62 THz. The RDP and PWP of the strip-shaped metamaterial can be determined quantitatively (detailed derivation is proposed in the Supplementary Materials).

**Figure 1:**
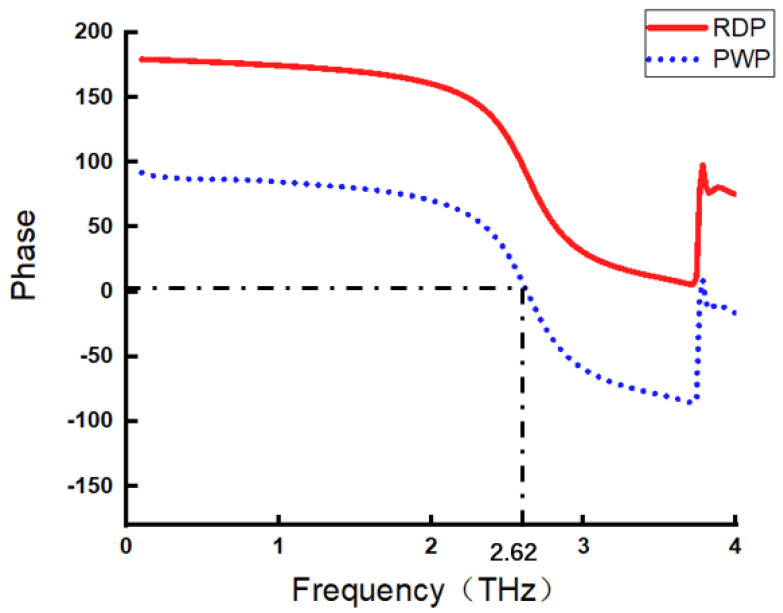
PWP and RDP curves vary with incident frequencies (resonance frequency is 2.62 THz).

Figure 1 shows the variation curves of the RDP and PWP at different incident photon frequencies. They exhibit the same trend of variation but a unit phase difference of 90 degrees.

When the incident photon frequency is relatively lower than the resonant frequency of the metamaterial, the PWP is positive. This means that the metamaterial will bring about a positive phase shift because the intrinsic frequency of the metamaterial is higher than that of the incident photon. However, when the incident photon frequency is higher than the intrinsic frequency, the PWP becomes negative. This means that the metamaterial will cause a negative phase shift because the response delay of the metamaterial will cause a lagging effect as it drags the photos along. The abnormal phase changes at the high-frequency band of the PWP curves are caused by non-geometric optical scattering, which is also a common principle for most metamaterial absorbers [12,13].

According to the definitions of RDP and PWP, it can be considered that near-field photons (with RDP) are the precursor of the far-field photons (with PWP). The photons with RDP emitted by the resonant dipole form around the array element atoms of the metamaterial and induce spatial coherence and phase alignment, resulting in far-field photons with PWP. The 90-degree phase difference between the RDP and PWP confirms their causal relationship. Energy also transitions from the near-field photons to the far-field photons: when the near-field photons reach their maximum amplitude, the far-field photon amplitude is zero and vice versa, which is in accordance with the law of energy conservation. In comparison to the RDP, the PWP is easier to measure to reveal the metamaterial’s response to incident photons.

## 3. Verification of the MIPRT on a Single-Layer Metamaterial

As mentioned above, energy conservation within MIPRT is consistent with photon interference in the transmitted half space and reflection in the incident half space. When the number of photons involved is sufficiently large, the photon description can be effectively replaced by a classical electric field representation. In this regime, the energy is strongly dependent on the field amplitude, whereas photon interference is predominantly governed by the phase difference of the electric field. Therefore, it can be anticipated that the field amplitude and phase are intrinsically linked.

**Figure 2:**
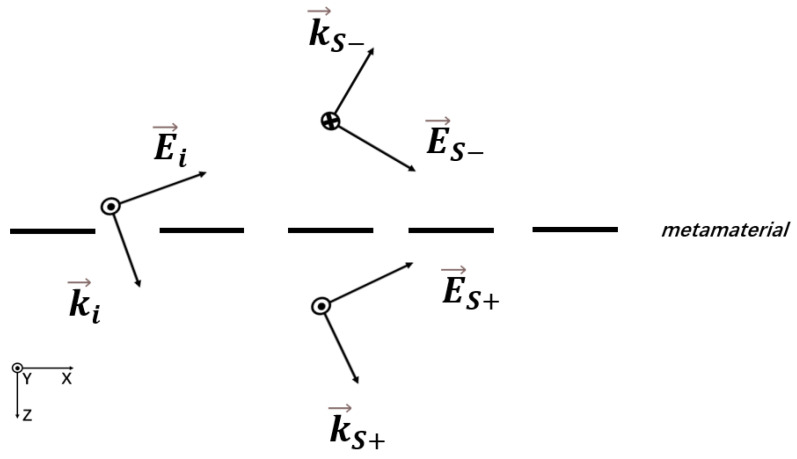
A schematic diagram of a single-layer metamaterial.

Applying MIPRT on a single-layer metamaterial, Figure 2 shows the diagram of the field decomposition, assuming that the incident electric field wavevector is ${\vec{k}}_{i}$ throughout the entire space. In the steady state, the excited resonant dipole in the metamaterial will radiate the electric field outward. The radiation electric field wavevector is ${\vec{k}}_{s}$, and it is represented as ${\vec{k}}_{s-}$ in the incident half space and ${\vec{k}}_{s+}$ in the transmitted half space with symmetric direction. More generally, for the case of multi-lobe radiation of the resonant dipole, the electric field wavevector of each lobe can be expressed as ${\vec{k}}_{s2}$, ${\vec{k}}_{s3}$, …, and the amplitude–phase relationship of Equation (2) can be obtained by solving Maxwell’s equations under the boundary conditions (detailed derivation is presented in the Supplementary Materials)

(2) $$\begin{array}{c} \frac{\sum_{m=1}^{n}\left[E_{sm0}\times cos\left(\alpha_{m}\right)\right]}{E_{i0}}=-cos\left(\phi \right)\;\;\;\;\;\left(n=1,2,3\ldots \right) \end{array}$$

where $E_{i0}$ is the electric field amplitude of the incident electric field, $E_{sm0}$ is the electric field amplitude of the $m^{th}$ radiation lobe whose electric field wavevector is ${\vec{k}}_{sm}$, $\alpha_{m}$ is the angle between ${\vec{k}}_{sm}$ and ${\vec{k}}_{i}$, and ϕ is the PWP.

Based on Equation (1), the definition of the PWP, and the electromagnetic field distribution shown in Figure 2, it can be concluded that the PWP in this case is equal to the phase of the reflected wave. In addition, further investigation within this model is expected to enable the derivation of the relationship between the transmitted wave phase and the aforementioned parameters.

For the simplest case, there is only one dipole radiation lobe; when thermal loss is negligible (the most common treatment for metal structural metamaterials), Equation (2) can be simplified as Equation (3):

(3) $$\begin{array}{c} R=cos^{2}\left(\phi \right) \end{array}$$

*R* is reflectivity of the metamaterial. Equation (3) indicates that the PWP and the reflectivity are strongly coupled and highly correlated, implying that the phase of the reflected wave directly determines its amplitude.

**Figure 3:**
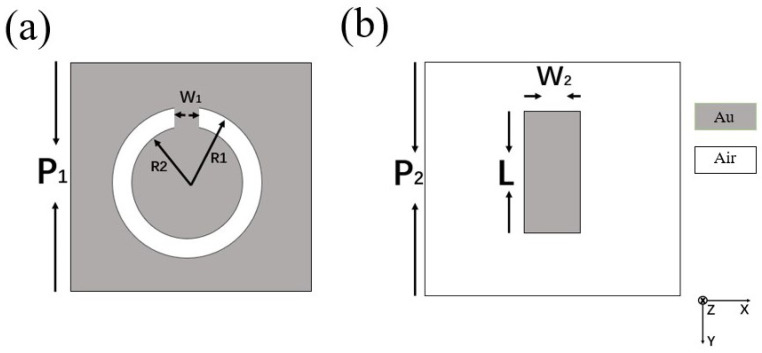
Unit structures of metamaterial. (**a**) Open ring slit unit with $P_{1}$ = 15 mm, $W_{1}$ = 1 mm, *R*1 = 3.5 mm, and *R*2 = 5 mm. (**b**) Long strip unit with $P_{2}$ = 80 μm, $W_{2}$ = 8 μm, and *L* = 50 μm.

Two typical metamaterial structures are randomly selected to verify the amplitude–phase relationship of Equation (3). One comes from the COMSOL 5.4 case library [14] with open ring slit unit structure, as shown in Figure 3a; Figure 3b is another one, which has long metal strip unit structures.

**Figure 4:**
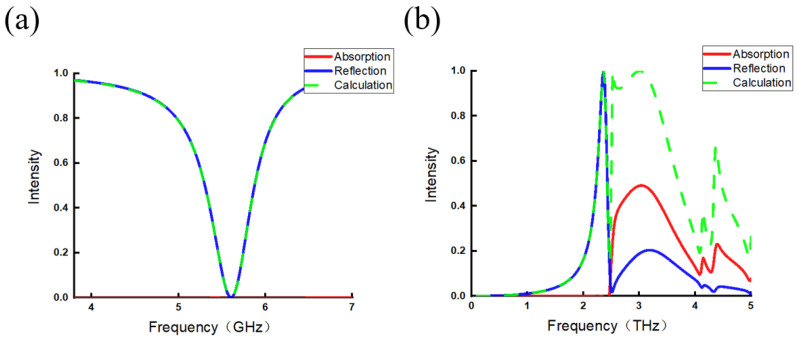
Simulated absorption curves (red line) of metamaterial structures and the comparison of reflection curves between the simulated (solid line) and theoretically calculated (dashed line) results. (**a**) Open ring slit. (**b**) Long strip.

The two metamaterial structures are incident by EM waves at the angles of 0° and 30°, respectively, and the polarization of the incident electric field is perpendicular to the opening of the open ring slit or parallel to the long edge of the metal strip (+Y axis direction); the simulated curves of reflection and absorption of them are shown in Figure 4.

In metamaterials, absorption is commonly defined in the context of absorbers. Since such absorbers are predominantly developed for radar-related applications, the analysis typically focuses on the transmitted and reflected waves, and the absorption is consequently defined as one minus the transmittance and reflectance. In a broader physical sense, however, absorption usually refers to the conversion of electromagnetic energy into other forms of energy within the device. The absorption calculation in metamaterials therefore mixes two distinct contributions: energy dissipated through scattering and energy lost through thermal dissipation. Only the latter corresponds to absorption in the conventional sense. To more accurately describe the physical nature of the calculated absorption, we distinguish these two contributions as scattering absorption and thermal loss absorption, respectively.

In the metamaterial system considered here, the metal is treated as an ideal conductor. As a result, the absorption obtained from the absorption spectrum corresponds purely to scattering absorption.

Figure 4a shows a zero scattering absorption curve (red line) across the entire 0–7 GHz frequency range, representing no scattering absorption for the open ring slit metamaterial, so Equation (3) is applicable in the entire frequency band. The simulated reflectivity curve (blue solid line) fits well with the theoretically calculated results (green dashed line).

However, for the metal strip metamaterial, as depicted in Figure 4b, the scattering absorption curve shows a more complex variation: the no scattering absorption frequencies are below 2.5 THz, rendering Equation (3) applicable to this structure only within a partial frequency range (0–2.5 THz). So the correspondence between the simulated and theoretically calculated results are limited to the no absorption frequency range of 0–2.5 THz, while a notable deviation appears when the frequency range surpasses 2.5 THz. Hence, Equation (3) is verified to be the effect for the no scattering absorption case. However, since Equation (2) contains electric field vectors of all radiation lobes, it is expected to solve the scattering absorption problem.

**Figure 5:**
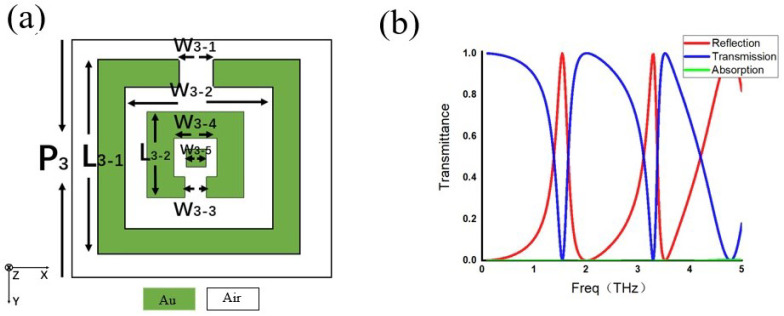
Meta-atom unit-cell geometry and transmission characteristics of the metamaterial. (**a**) $P_{3}$ = 40 μm, $W_{3-1}$ = 4.8 μm, $W_{3-2}$ = 20.4 μm, $W_{3-3}$ = 2.4 μm, $W_{3-4}$ = 10.2 μm, $W_{3-5}$ = 4 μm, $L_{3-1}$ = 30 μm, and $L_{3-2}$ = 15 μm. (**b**) Transmission, reflection, and absorption spectra of the metamaterial.

In the preceding sections, metamaterials with split-ring slit geometries and simple bar-shaped structures were discussed. In what follows, Equation (3) is further verified using a more complex metamaterial configuration. The corresponding meta-atom geometry and its transmission characteristics are shown in Figure 5:

Figure 5a illustrates the meta-atom structure of this metamaterial, where the green regions denote metal and the white regions denote free space. Owing to the geometric complexity of the structure, multiple resonant features are expected to appear within the frequency spectrum. This expectation is confirmed in Figure 5b, where the red curve represents reflection, the blue curve represents transmission, and the green curve represents absorption. It can be observed that transmission minima coincide with reflection maxima, while absorption remains negligible over the frequency range of interest (0–5 THz).

**Figure 6:**
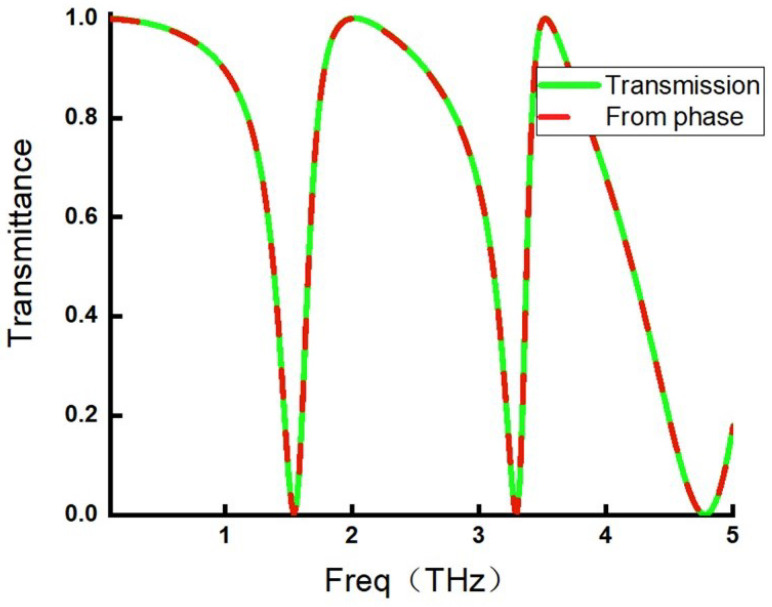
Comparison between the phase-calculated transmission spectrum and the actual transmission spectrum.

According to Equation (3), the reflection can be calculated from the phase of the resonant polariton, and the transmission spectrum can then be obtained accordingly. A comparison between the transmission spectrum calculated from the polariton phase and the directly simulated transmission spectrum is shown in Figure 6.

In Figure 6, the solid green line represents the directly obtained transmission spectrum, while the dashed red line corresponds to the transmission spectrum calculated from the phase. The two curves exhibit excellent agreement over the entire frequency range, demonstrating the validity of the phase-based transmission calculation. This result directly confirms the correctness of Equation (3) and the MIPRT framework.

**Figure 7:**
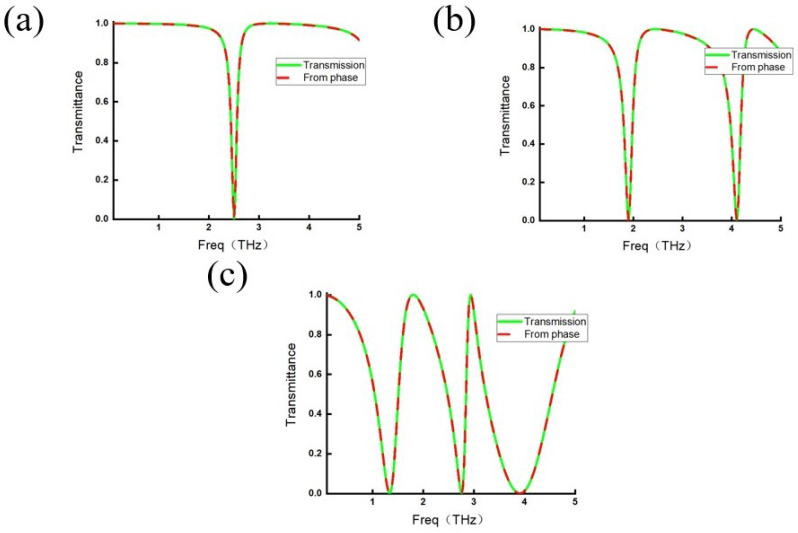
Comparison between the phase-calculated and actual transmission spectra for different scaling factors. (**a**) Scaling factor of 0.6. (**b**) Scaling factor of 0.8. (**c**) Scaling factor of 1.2.

To examine whether this agreement is merely coincidental for a specific choice of structural parameters, the overall dimensions of the metamaterial are uniformly scaled by factors of 0.6, 0.8, and 1.2. The corresponding comparisons between the calculated and actual transmission spectra are presented in Figure 7. Figure 7a shows the case with a scaling factor of 0.6, where only a single transmission dip appears within the 0–5 THz range and the calculated transmission curve remains consistent with the actual one. In Figure 7b, corresponding to a scaling factor of 0.8, two transmission dips emerge, and again the calculated and actual transmission spectra coincide well. For the scaling factor of 1.2 shown in Figure 7c, the enlarged meta-atom gives rise to three transmission dips within the considered frequency range, and the calculated transmission curve matches the actual transmission spectrum across the entire band.

In summary, for this geometrically complex metamaterial, the transmission spectra calculated from the resonant polariton phase consistently agree with the actual transmission spectra across multiple structural scaling parameters. This demonstrates that the observed agreement is not accidental but instead reflects the intrinsic validity of Equation (3) and the MIPRT framework. Combined with the results obtained for the two simpler metamaterial configurations discussed earlier, these findings indicate that MIPRT successfully describes electromagnetic wave modulation in simple and complex metamaterials, slit-based structures, and cases with nonzero incidence angles. The amplitude–phase relationships revealed by Equation (3) are consistently satisfied, further indicating that MIPRT provides a reliable theoretical framework for uncovering the underlying physical principles governing electromagnetic modulation in metamaterials.

Although the present study is limited to numerical validation and does not include experimental cross-verification, the validation performed is sufficiently comprehensive. It is well established that, when properly configured, numerical simulations show good agreement with experimental results. In this work, the proposed theory is verified across multiple distinct metamaterial configurations, including representative structures drawn from the simulation software’s benchmark library, which further confirms the correctness of the simulation settings. These results collectively indicate that experimental validation is not essential for supporting the conclusions of this study.

Nevertheless, future experimental verification, if pursued, could be readily achieved by extracting the phase and amplitude of the reflected wave to directly test the validity of Equation (3). Alternatively, the theory could be examined by retrieving the resonant phase of the additional resonant dipolar field associated with the meta-atoms, although extracting near-field phase and field intensity experimentally remains technically challenging.

In addition, all metamaterial structures considered in this work are assumed to be ideally infinite and periodic. While practical experiments are necessarily limited to finite-sized samples, sufficiently large finite metamaterials can be well approximated as infinite when illuminated by realistically finite sources. Therefore, the proposed model and conclusions remain applicable to finite-sized metamaterial implementations under such conditions.

## 4. Indirect Verification via the Extinction Theorem

In optical theory, there exists a fundamental result known as the extinction theorem, first established by Ewald [15] and Oseen [16]. This theorem states that, at any point inside a medium, the incident wave is extinguished through destructive interference with the dipole fields induced in the medium and is replaced by another wave that propagates with a different phase velocity. It reveals that the dipole field inside a medium can be regarded as the superposition of a negative incident field and a secondary wave with a modified propagation speed.

When the medium is replaced by a metamaterial, this concept becomes distorted: the dipoles in the medium are no longer elementary material dipoles but instead transform into the resonant dipoles discussed in the previous sections. Consequently, the essence of the extinction theorem is intrinsically embedded in the MIPRT. In this section, we provide a derivation based on this perspective.

We begin by expressing the fundamental form of the extinction theorem [17] using Green’s function $G(r,\;r^{\prime })$:

(4) $$\begin{array}{c} \int G\left(r,\;r^{\prime }\right)\left[-i\omega P(r^{\prime })\right]d^{3}r^{\prime }=-{\vec{E}}_{i}\left(r\right)+{\vec{E}}_{med}\left(r\right) \end{array}$$

where *r* denotes the observation point, $r^{\prime }$ the source point, ${\vec{E}}_{i}\left(r\right)$ the incident field, ${\vec{E}}_{med}\left(r\right)$ the field inside the medium, ω the angular frequency, and $P(r^{\prime })$ the electric dipole moment density of the medium.

When this formulation is applied to metamaterials, the left-hand side of the equation represents the superposition of radiation emitted by the resonantly excited resonant dipoles:

(5) $$\begin{array}{c} \sum G_{meta}\left(r,\;r^{\prime }\right)\left(-i\omega \right)p\left(r^{\prime }\right)=-{\vec{E}}_{i}\left(r\right)+{\vec{E}}_{med}\left(r\right) \end{array}$$

For a single-layer metamaterial, we assume that the meta-atoms are distributed periodically on the plane z=0. Under this assumption, the spatial and temporal radiation patterns of each meta-atom are identical and differ only by a phase shift determined by their reference position. This leads to

(6) $$\begin{array}{c} \left(-i\omega \right)p_{0}\sum G_{meta}\left(r,\;r^{\prime }\right)e^{jk_{\left|\right|}\cdot \left(r^{\prime }-r\right)}=-{\vec{E}}_{i}\left(r\right)+{\vec{E}}_{med}\left(r\right) \end{array}$$

(7) $$\begin{array}{c} k_{\left|\right|}^{2}=k_{i}^{2}-k_{z}^{2} \end{array}$$

where the subscript || denotes the direction parallel to the metamaterial surface and $p_{0}$ is a constant representing the resonant electric dipole moment of a meta-atom.

We now examine the modulation of the electromagnetic wave by the metamaterial. Since the observation point is taken to be in the far field, the radiation term on the left-hand side can be decomposed into a plane wave propagating along the principal radiation direction $\left(A_{0}e^{j{\vec{k}}_{main}\cdot \vec{r}}\right)$ and higher-order diffraction components ${\vec{E}}_{D}\left(r\right)$, where $A_{0}$ is a constant. Thus,

(8) $$\begin{array}{c} A_{0}e^{j{\vec{k}}_{main}\cdot \vec{r}}+{\vec{E}}_{D}\left(r\right)=-{\vec{E}}_{i}\left(r\right)+{\vec{E}}_{t}\left(r\right) \end{array}$$

According to the definition of resonant dipole radiation introduced earlier, we have

(9) $$\begin{array}{c} {\vec{E}}_{s}\left(r\right)=A_{0}e^{j{\vec{k}}_{i}\cdot \vec{r}}+{\vec{E}}_{D}\left(r\right) \end{array}$$

Substituting this expression into Equation (8), we obtain

(10) $$\begin{array}{c} {\vec{E}}_{t}\left(r\right)={\vec{E}}_{i}\left(r\right)+{\vec{E}}_{s}\left(r\right) \end{array}$$

This equation constitutes the electric field representation of Equation (1). It indicates that, in the transmission region, there exists a virtual incident field component. After coherent destructive interference between this virtual field and the radiation emitted by the meta-atoms, the resulting field manifests as the transmitted wave. In this manner, the physical meaning of the extinction theorem is incorporated into the metamaterial framework, and the validity of MIPRT is verified through the concept of extinction.

## 5. Summary and Conclusions

This paper aims to provide a clear and concise insight into the interaction between metamaterials and incident photons. Based on the Invariant Incident Photon Hypothesis and Resonant Phase Deconstruction and Quantification, MIPRT is proposed to clarify the complex modulation process of metamaterials on incident photons. This theory assumes the coexistence of invariant incident photons and resonant dipoles within the metamaterial; then photons emitted from the resonant dipoles will coherently interact with incident photons. The PWP and RDP are defined and quantified to describe the resonant phase modulation process of metamaterials on incident photons. The theoretical relationship between the PWP and reflectivity of the metamaterials is derived by applying MIPRT on a single-layer metamaterial model; the results are verified by simulation.

The MIPRT introduces a new metamaterial research approach and is expected to provide a more detailed and reasonable physical explanation for the interaction between metamaterials and incident photons. In addition, the development of theoretical frameworks can effectively facilitate the expansion of potential applications. In micro- and nanoscale devices, the present theory reveals that the amplitude and phase responses obey trigonometric relationships. This feature suggests that such devices may enable nonlinear functional modulation through appropriate transformations in optical modulation processes. Consequently, this mechanism indicates potential applicability in photonic neural network architectures, where it could serve a role analogous to an activation function.

## Data Availability

The data that support the findings of this study are available from the corresponding author upon reasonable request.

## Supplementary Materials

The following supporting information can be downloaded at: https://www.mdpi.com/article/10.3390/mi17010130/s1.
