# Supplementary material for: Metamaterial Incident Photon Reconstruction Theory Based on Resonant Dipole Phase
Source: Micromachines (Basel). 2026 Jan 20;17(1):130. doi: 10.3390/mi17010130 (PMC12843878; doi:10.3390/mi17010130)
Supplement: Supplementary file 1 [file micromachines-17-00130-s001.zip › micromachines-4072736-supplementary.pdf]

Supplementary for Metamaterial Incident Photons Reconstruction Theory Based on Resonant Dipole Phase

Boli Xu and Renbin Zhong*

Terahertz Research Center, School of Electronic Science and Engineering, University of Electronic Science and Technology of China, Cooperative Innovation Centre of Terahertz Science, Chengdu 610054, P.R. China

E-mail: rbzhong@uestc.edu.cn

Supplementary

Supplementary: Detailed calculation on RDP and PWP

With Invariant Incident Photon Hypothesis, we can understand the impact of the metamaterial on the total field distribution by focusing our discussion only on the REM. To explore the resonant phase definition of REM, we take a long strip-shaped metamaterial as a case, with the strip length of 50 μm and a period of 80 μm, its resonance frequency is 2.62 THz. According to Equation (1), subtracting the incident field from the total field, Fig. S1. shows the REM field distribution in the time domain when it is incident by 2.62 THz wave. A strong near field is excited around meta-atom, and forms the resonant dipole (marked by the black frame).

To study the PWP, considering the reflected wave (only REM included) with electronic field distribution shown in Fig. S2(a), we focus on the variation of the electric field amplitude

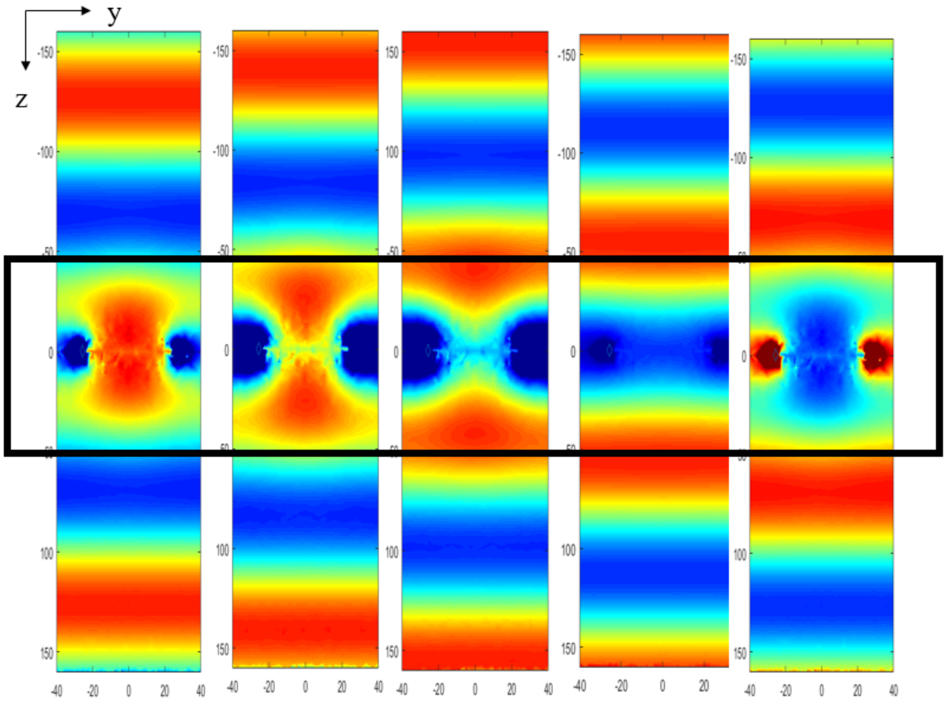

Figure S1: Diagram of resonance dipole field variation in time domain

along the Z axis direction at y=1 (referred by the black line). The solid curve in Fig S2(b) plots the variation of electric field amplitude of the reflected wave. The PWP can be determined quantitatively by comparing it with a standard sine curve of zero-phase (the doted curve in Fig. S2(b)), well-fit of the two curves, indicates that the PWP is zero at the case of 2.62 THz incidence. So, the incident EM wave is only reflected by the metamaterial without additional phase shift.

For the case of incident frequency is 1.5 THz, which is lower than the resonance frequency of the strip-shaped metamaterial, as shown in Fig. S3(a). We still extract the amplitude values of electric field along the black line, and the variation curve is plotted in Fig. S3(b). 80 degrees delay of PWP can be found, by fitting a standard sine waveform. Similarly, when the metamaterial is incident by the EM wave with a higher frequency of 3.26 THz, we observe that the delay of PWP is -72 degrees, as depicted in Fig S4. Consequently, we can obtain the curve of PWP changing with the frequency of the incident wave, which is shown as dotted curve in Fig. 5.

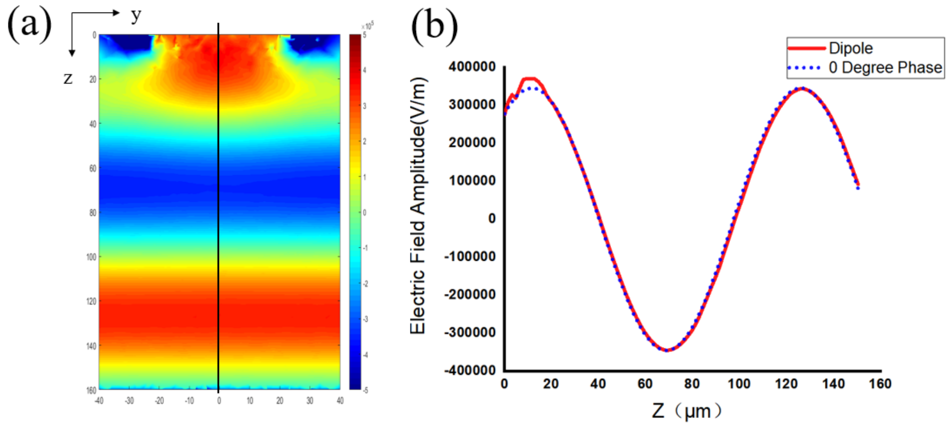

Figure S2: (a) The field diagram of REM in the incident space. (b) The amplitude of the electric field variation along the black line of perpendicular of the resonant dipoles.

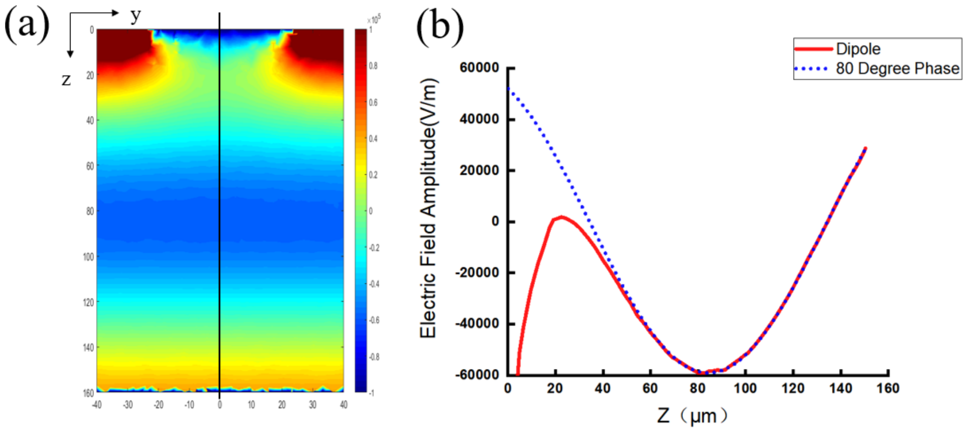

Figure S3: (a) The field diagram of REM in the incident space. (b) The variation of the amplitude of the electric field along the black line.

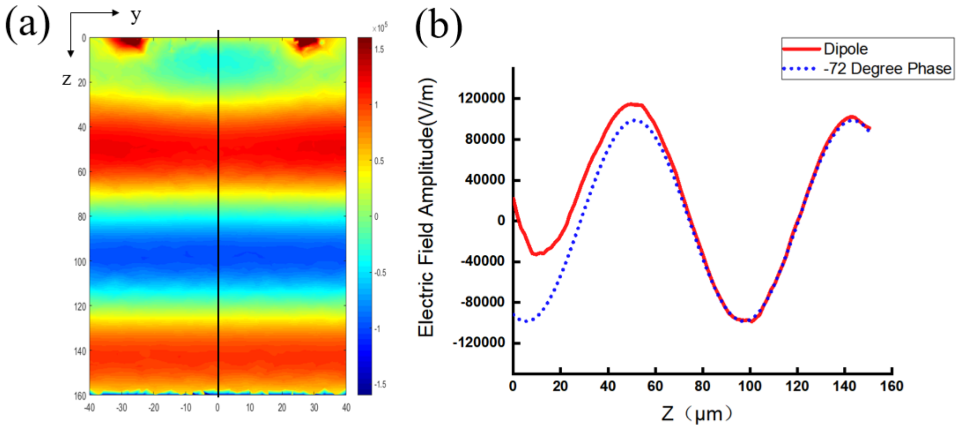

Figure S4: (a) The field diagram of REM in the incident space. (b) The variation of the amplitude of the electric field along the black line.

The RDP of the resonance dipoles of the strip-shaped metamaterial can be determined quantitatively in time domine in the same method. In Fig. 5, the solid curve shows the variation of the RDP at different incident EM wave frequencies.

Development of a Single-layer Metamaterial Model

Then, the physical model of the excited metamaterial surface is established as shown in Fig. S5, the metasurface is on the XOY plan between mediums air 1 and air 2, when it is normal incident by a plane EM wave $\overrightarrow{E}_i$ from upper half space, surface current J is excited, and radiates uniform EM wave $\overrightarrow{E}_{s1}$ and $\overrightarrow{E}_{s2}$ to air 1 and air 2 respectively at a steady state. Assume $\overrightarrow{E_{s1}}$ is parallel electric field component along +X direction, its wave vector $\overrightarrow{k}_{s1}$ is along -Z direction, correspondingly, $\overrightarrow{E}_{s2}$ in air 2 is along +X direction with $\overrightarrow{k}_{s2}$ in +Z direction. EM fields $\overrightarrow{E}_i$ in air 2 is the same as that in air 1, with $\overrightarrow{k}_i$ in +Z direction, which is consistent to the physical mechanism. Thus, in this model, we can regard $\overrightarrow{E}_{s1}$ as the reflected electric field in air 1 and $\overrightarrow{E}_i + \overrightarrow{E}_{S2}$ is the total transmitted EM wave in air 2.

Assume $e^{j\omega t}$ is time factor, and the phase of $\overrightarrow{E}_i$ on the XOY plane is zero. Then, the EM field vectors on both sides of the current layer can be expressed as follows:

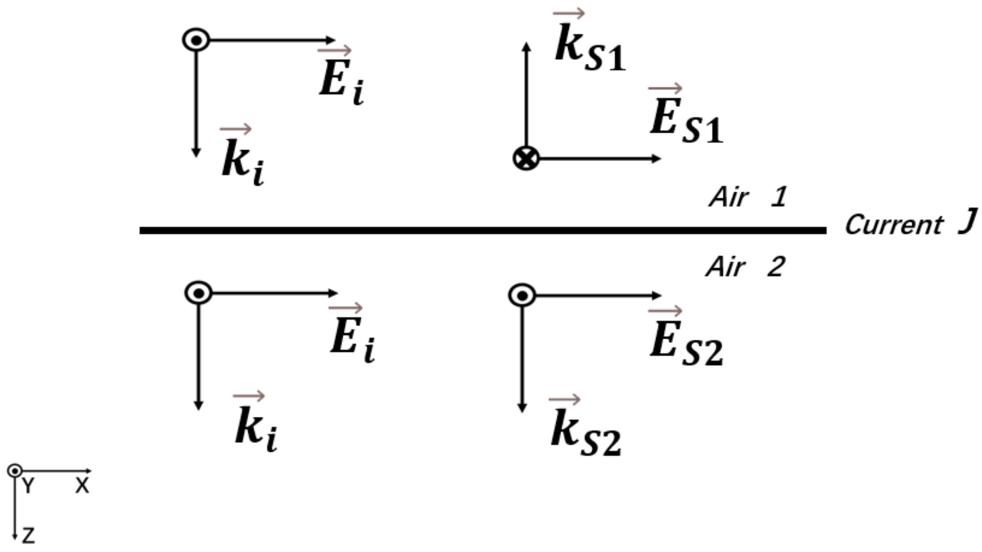

Figure S5: Model diagram.

$$\overrightarrow{E}_i = \overrightarrow{e}_x E_{i0} e^{jkz-j\omega t} \tag{S1}$$

$$\overrightarrow{H}_i = \overrightarrow{e}_y \frac{E_{i0}}{\eta_0} e^{jkz-j\omega t} \tag{S2}$$

$$\overrightarrow{E}_{S2} = \overrightarrow{E}_{S0+} e^{-j\omega t} e^{j\phi} \tag{S3}$$

$$\overrightarrow{H}_{S2} = \overrightarrow{e}_y \frac{\sum_{m=1}^{n}[E_{sm0}e^{jkr_+}]}{\eta_0} e^{-j\omega t} e^{j\phi} \tag{S4}$$

$$\overrightarrow{E}_{S1} = \overrightarrow{E}_{S0-} e^{-j\omega t} e^{j\phi} \tag{S5}$$

$$\overrightarrow{H}_{S1} = -\overrightarrow{e}_y \frac{\sum_{m=1}^{n}[E_{sm0}e^{jkr_-}]}{\eta_0} e^{-j\omega t} e^{j\phi} \tag{S6}$$

$$\overrightarrow{E}_{S0+} = \sum_{m=1}^{n} [[E_{sm0} \times cos(\alpha_m)]\overrightarrow{e}_x + [E_{sm0} \times sin(\alpha_m)]\overrightarrow{e}_y] e^{-jkr_+} \tag{S7}$$

$$\overrightarrow{E}_{S0-} = \sum_{m=1}^{n} [[E_{sm0} \times cos(\alpha_m)]\overrightarrow{e}_x + [E_{sm0} \times sin(\alpha_m)]\overrightarrow{e}_y] e^{-jkr_-} \tag{S8}$$

$$r_+ = cos(\alpha_m)z + sin(\alpha_m)x \tag{S9}$$

$$r_- = -cos(\alpha_m)z + sin(\alpha_m)x \qquad (n = 1, 2, 3...) \tag{S10}$$

Here, η_0 represents the impedance of free space, other parameters subscripted with 0 stand for the amplitudes, and ϕ indicates the additional phases that may occur after time zero. E_{sm0} and α_m represent the electric field magnitude and direction of the mth wavefront, respectively, with the electric field direction being the angle between the x-axis and the electric field.

According to equation (S1) - (S10), the total fields of the model are $\overrightarrow{E}_1$ and $\overrightarrow{H}_1$ in air 1, $\overrightarrow{E}_2$ and $\overrightarrow{H}_2$ in air 2:

$$\overrightarrow{E}_1 = \overrightarrow{E}_i + \overrightarrow{E}_{S1} \tag{S11}$$

$$\overrightarrow{H}_1 = \overrightarrow{H}_i + \overrightarrow{H}_{S1} \tag{S12}$$

$$\overrightarrow{E}_2 = \overrightarrow{E}_i + \overrightarrow{E}_{S2} \tag{S13}$$

$$\overrightarrow{H}_2 = \overrightarrow{H}_i + \overrightarrow{H}_{S2} \tag{S14}$$

$\overrightarrow{E}_{s1}$ and $\overrightarrow{E}_{s2}$ is radiated by surface current $\overrightarrow{J}$, following the physical principles of surface current radiation:[1]

$$\overrightarrow{n} \times (\overrightarrow{H}_{S2} - \overrightarrow{H}_{S1}) = \overrightarrow{J} \tag{S15}$$

Equation (S15) can be transformed to the equation (S16) of $\overrightarrow{H}_1$ and $\overrightarrow{H}_2$ by substituting with equations (S11) - (S14):

$$\overrightarrow{n} \times (\overrightarrow{H}_2 - \overrightarrow{H}_1) = \overrightarrow{J} \tag{S16}$$

Obviously, equation (S16) conforms naturally to the boundary condition of the total magnetic fields in both sides of the constructed metasurface mode.

From the conventional perspective of energy conservation law, it is assumed that the electric field of incident wave is i=$\overrightarrow{E}_i$; electric field of transmitted wave is t=$\overrightarrow{E}_2$ and reflected electric field is r=$\overrightarrow{E}_{s1}$. Then the energy flow can be expressed as: $I = C_0 i \cdot i^*$, $T = C_0 t \cdot t^*$, $R = C_0 r \cdot r^*$, respectively. C_0 represents a constant. With the ignorable loss of the current layer, the equation of conservation of energy is well known as:

$$T + R = I \tag{S17}$$

Substituted with equations (S1) - (S14), equation (S17), the amplitude-phase relationship of the reflected wave modulated can be attained:

$$\frac{\sum_{m=1}^{n}[E_{sm0} \times cos(\alpha_m)]}{E_{i0}} = -cos(\phi) \qquad (n = 1, 2, 3...) \tag{S18}$$

References

(1) COMSOL Multiphysics. *version 2018*
